# A Novel Rat Tail Needle Minimally Invasive Puncture Model Using Three-Dimensional Printing for Disk Degeneration and Progressive Osteogenesis Research

**DOI:** 10.3389/fcell.2021.587399

**Published:** 2021-06-03

**Authors:** Dongdong Xia, Meijun Yan, Xin Yin, Wenhao Hu, Chi Zhang, Baiwen Hu, Ting Ge, Xiaochuan Wu, Jin Xiao, Liang Gao, Junqi Liu, Jun Tan

**Affiliations:** ^1^Department of Orthopedics, Ningbo First Hospital, Ningbo, China; ^2^Department of Orthopaedics, Shanghai General Hospital, Shanghai, China; ^3^Department of Orthopedics, Fourth Medical Center of PLA General Hospital, Beijing, China; ^4^Center for Clinical Medicine, Hua Tuo Institute of Medical Innovation (HTIMI), Wuhan, China; ^5^Department of Radiotherapy, The First Affiliated Hospital of Zhengzhou University, Zhengzhou, China; ^6^Department of Orthopedics, Shanghai East Hospital, School of Medicine, Tongji University, Shanghai, China

**Keywords:** disk degeneration, rodent tail model, vertebral hyperosteogeny, minimally invasive procedure, 3D printing technique

## Abstract

Many studies focused on the annulus fibrosus (AF) injury in rodent tail model for the intervertebral disk degeneration (IDD) research. However, previous studies caused tremendous injury of intervertebral disk (IVD) by penetrating whole disk. This study aimed to build a progressive IDD rodent tail model by a novel device for precise and minimally invasive puncture in AF. A precise puncture device was customized by 3D Printing Technique. 40 rodent tail IVDs were randomly grouped as follows: group A, non-puncture; group B, annulus needle puncture (ANP) for 4 week; group C, ANP for 8 week; and group D, ANP for 12 week. Pre- and post-puncture IVD height on radiographs and IVD signal intensity on T2 magnetic resonance imaging (MRI) were measured. Average bone density (ABD) on the end of coccygeal vertebrae between punctured disk was measured on the radiographs. Hematoxylin and eosin, TUNEL staining methods, immunofluorescence for cleaved-caspas3 and immunohistochemistry for aggrecan and collagen II were performed. Progressively and significantly increasing IVD height loss and degenerative grade were observed following the time points. The ABD was respectively, 81.20 ± 4.63 in group A, 83.93±3.18 in group B, 92.65 ± 4.32 in group C, 98.87 ± 6.69 in group D. In both group C and group D, there were significant differences with group A. In histology, increasing number of AF cells was noted in group B. In both group C and D, the fissures in AF were obviously observed, and a marked reduction of AF cells were also observed. In all ANP groups, there were significant decrease in number of NP cells, as well as aggrecan and collagen II contents. TUNEL assay showed cellular apoptosis were stimulated in all puncture group, especially in group D. A progressive IDD rat model could be standardly established by the micro-injury IVD puncture using a novel 3D printing device. This animal model provided a potential application for research of progressive hyperosteogeny following IDD development.

## Introduction

Intervertebral disk degeneration (IDD) is a common pathophysiological condition in humans affecting the intervertebral disc (IVD), which can include height decrease, disk herniation, and vertebral osteogenesis, due to its implication in causing lower back pain (Deyo and Weinstein, 2001; Alini et al., 2008). As animal IVD presents a similar mechanical and biochemical environment to human IVD (Adams and Roughley, 2006), many animal models have been developed to provide a reliable guide to the study of biologic processes in degenerating disks. To mimic the degeneration process in human IVD, different *in vivo* animal models were built as mechanical type (Kroeber et al., 2002; Bergknut et al., 2012; Xia et al., 2015), chemical type (Ando et al., 1995; Yamada et al., 2001; Norcross et al., 2003), spontaneous type (Kimura et al., 1996; Watanabe et al., 1997; Sahlman et al., 2001), or injury type (Key and Ford, 1948; Smith and Walmsley, 1951; Lipson and Muir, 1980). A new method of annulus needle puncture (ANP) rabbit model was reported to establish a reproducible animal model of disk degeneration that could be detected in slowly progressive changes by magnetic resonance imaging (MRI), radiographs, and histology (Masuda et al., 2005; Sobajima et al., 2005). However, an incision was needed in the rabbit ANP model to explore the disk from the skin and muscles, which increased the risk of damage to surrounding structures and was time-consuming.

With minimal risk of damage to surrounding structures and minimal interference with normal physiological function, the rat tail discs (RTD) were more suitable and feasible for the ANP method to induce IDD (Han et al., 2008; Issy et al., 2013; Grunert et al., 2014; Liao, 2016; Cunha et al., 2017). However, there were still some limitations of RTD models in previous studies. According to our preliminary study, the width of the RTD was only about 1 to 2 mm. Most of the reported studies used the 18G (diameter = 1.2 mm) or 21G (diameter = 0.8 mm) needle to puncture the RTD, which lacked the meaning of progressive degeneration research due to great injury to the IVD. Moreover, there was no specific protocol to standardize how to puncture the disk, such as the puncture direction and the puncture point. An improper puncture point may cause hemorrhage and extra trauma due to neglecting the position of four main vessels in the tail. There was also no uniform standard for the depth of the puncture.

Thus, the purpose of this study is to establish a novel minimally invasive ANP rat model using customized precise puncture devices by three-dimensional (3D) printing technique. Under the standardized protocols of puncture point and depth, we can induce a progressive IDD model in a few-minute procedure without extra bleeding. MRI and histological studies were performed to evaluate the progression of IDD.

## Materials and Methods

### Animals

Forty skeletally mature Sprague Dawley (SD) rats (3 months old) weighing an average of 350 g (purchased from the Center for Laboratory Animals, Ningbo University) were used in this study. Animal manipulation was conducted in compliance with Chinese legislation regarding the care and use of laboratory animals and approved by the Animal Care and Use Committee of Ningbo University.

### Animal Groups

In this experiment, the rats were randomly assigned into four groups: one non-puncture group (*n* = 10) and three ANP groups for 4, 8, and 12 weeks, respectively (*n* = 10 for each group) (Table 1). In the ANP groups, a customized 3D-printing puncture device was used to puncture the RTD in the Co7/8 segment. The animals in the non-puncture group served as intact control subjects. Before the experiment, all the rats were given several days to adapt to the new housing and husbandry environment.

**TABLE 1:** Groups for Animal Surgery.

**Group**	**N**	**Type of group**	**Description of treatment**
A	10	Sham-control	No puncture surgery
B	10	Experimental	Puncture surgery for 4 weeks
C	10	Experimental	Puncture surgery for 8 weeks
D	10	Experimental	Puncture surgery for 12 weeks

### ANP Device

The novel device for ANP was customized by 3D printing technique for the precise puncture purpose. Based on the measurement of rat tail dimensions, the 3D image of the tail was reconstructed by UG 8.5 (Siemens, Germany). The size and shape of the ANP device were designed as a ring that could exactly match the experimental level of the rat tail. The device consisted of two parts: the ring body with eight holes which were used for puncture and localization and the needle holders inserted with a 27G needle (Becton Dickinson, United States) in a certain length. The needle holders firmly fitted the puncture holes from four directions of the ring body to control the puncture depth under the requirement. In our preliminary research, the width of the nucleus pulposus (NP) was measured as less than 5 mm at the punctured level (Co7/8) no matter how the whole tail width varies. Thus, we controlled the needle tip gap between each side in 5.5 mm by the needle holder, which ensured that all punctures were made into the annulus without penetrating the NP despite the variation of tail width. The device was designed by Autodesk 123D (California, United States) and produced with Nylon PA2200 in EOS Formiga P110 3D Printer (Germany).

### ANP Procedure

**FIGURE 1:**
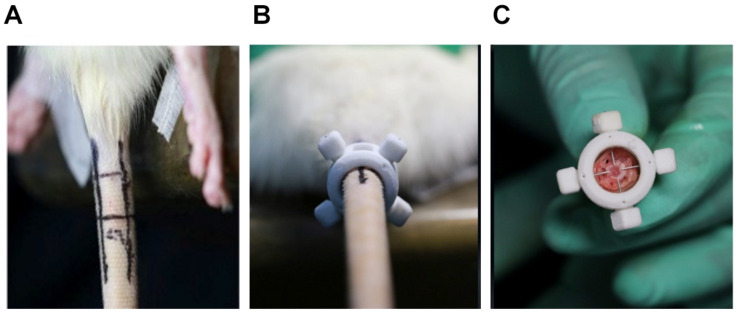
**(A)** Under digital palpation guidance, rat tail discs (Co7/8) and four main vessels were noted with a permanent marker. **(B)** The annulus needle puncture device body was attached to the rat tail, and 27G needles were used to puncture the annulus fibrosus (AF) though four localization holes. **(C)** During the puncture operation, 27G needles penetrate the entire layer of the AF without directly destroying the nucleus pulposus.

For the procedure preparation, the rats were weighed and injected intraperitoneally with 4% chloral hydrate at a dose of 0.4 ml/100 g body weight. As described in a previous study, the experimental level RTD (Co7/8) was located by digital palpation on the coccygeal vertebrae and confirmed by counting the vertebrae from the sacral region in a trial radiograph (Han et al., 2008). The punctured disk level was determined by palpation and four main vessels confirmed by direct vision were marked on the skin surface (Figure 1A). Then, the customized ANP device ring body was attached to the tail with the disk level seen in the middle of the puncture holes and the four vessels’ marker observed through each localization hole so that the vessels’ injury could be prevented during the puncture procedure under real-time monitoring (Figure 1B). After the ring body was attached, 27G needles were used to puncture the whole layer of the annulus fibrosus (AF) by needle holders matching the puncture holes (Figure 1C). To control the puncture depth, the length of the needle was customized according to preliminary measurements.

Daily monitoring of the rats was carried out to ensure their well-being, and all animals were allowed free unrestricted weight bearing and activity. After 4, 8, and 12 weeks of puncture, the animals were given X-ray and MRI examinations. After the X-ray and MRI examination, the animals were euthanized by an excess dose of chloral hydrate intraperitoneal injection, and the experimental RTD was excised for histological examination.

### Radiology and Image Analysis

Radiographs of the rat tails were taken before and at 4, 8, and 12 weeks after puncture. Before imaging, the animals were anesthetized as indicated above. To obtain a similar degree of muscle relaxation, which may affect the disk width [4], the rats were anesthetized carefully with a precise dosage according to their body weight, and all radiographs were taken at consistent time intervals after injection. After inducing with anesthesia, the rats were placed in a prone position on a molybdenum target radiographic image unit (ZS-1001, Shimadzu Corp., Kyoto, Japan), with their tails laid straight on the platform. Radiographs were obtained using the image unit with a collimator-to-film distance of 66 cm, exposure of 63 mAs, and penetration power of 35 kV as described before [6]. After radiography, an orthopedic researcher performed all measurements in a blinded fashion. The average IVD width index (DWI) was calculated by averaging the measurements obtained from the ventral, middle, and dorsal portions of the IVD and dividing that by the average of the adjacent vertebral body width based on the method of Masuda et al. (2005). The region of interest at the end of the coccygeal vertebrae between the punctured disk was the same size for each rat, to measure the average bone density (ABD). Bone density was also presented in a heat map edited from original radiographs by Photoshop CC 2015 (Adobe, San Jose, CA, United States).

### Magnetic Resonance Imaging

Magnetic resonance imaging was performed to evaluate the signal and structural changes in sagittal T2-weighted images using a 3.0-T clinical magnet (Philips Intera Achieva 3.0MR). T2-weighted sections in the sagittal plane were obtained in the following settings according to previous studies: fast spin echo sequence with time to repetition of 5,400 ms and time to echo of 920 ms, 320 h × 9,256 v matrix, field of view of 260, and four excitations. The section thickness was 2 mm with a 0-mm gap. The MRIs were evaluated by another blinded orthopedic researcher using the classification of intervertebral disk degeneration as reported by Pfirrmann et al. (2001) (Table 2) (one point = grade I, two points = grade II, three points = grade III, and four points = grade IV).

**TABLE 2:** Definition of a Magnetic resonance imaging Grading Scale.

**Grade**	**Structure**	**Distinction of NP and AF**	**Signal intensity**	**Height of intervertebral disk**
I	Homogeneous, bright white	Clear	Hyperintense, isointense to CSF	Normal
II	Inhomogeneous with or without horizontal bands	Clear	Hyperintense, isointense to CSF	Normal
III	Inhomogeneous, gray	Unclear	Intermediate	Normal to slightly decreased
IV	Inhomogeneous, gray to black, decrease	Unclear	Intermediate to hypointense	Normal to moderately decreased

### Tissue Harvesting and Histology

The experimental RTDs with adjacent vertebral bodies were harvested and fixed with 10% formalin for 24 h at 4°C. The samples were then rinsed with phosphate-buffered saline (PBS) and decalcified in 10% (w/v) sodium citrate/22.5% (v/v) formic acid (Morse’s solution) for 10 days, neutralized with 5% sodium sulfate for 4 days, and washed with water for 4 days. The samples were then dehydrated, embedded in paraffin, and sectioned (5 μm). After heating, deparaffinization, and hydration, sagittal sections of each IVD were made at 5–7 lm intervals, placed on silane-coated slides, and stained with hematoxylin and eosin (H&E).

### TUNEL Staining

DNA fragmentation was detected by using an *in situ* Cell Death Detection Kit (Roche, South San Francisco, CA, United States). After being fixed with 4% paraformaldehyde for 1 h, the cells were incubated with 3% H_2_O_2_ and 0.1% Triton X-100 for 10 min. The cells were then washed with PBS and co-stained with TUNEL inspection fluid and 4′,6-diamidino-2-phenylindole. Three random microscopic fields per slide were observed under a fluorescence microscope (Olympus Inc., Tokyo, Japan).

### Immunohistochemical Analysis

Immunohistochemistry (IHC) staining was performed with EXPOSE rabbit-specific HRP/DAB detection IHC kit (rabbit antihuman polyclonal antibodies, Beijing Zhongshan Golden Bridge Biotechnology Co., Ltd., Beijing, China) according to the manufacturer’s instructions. Primary antibodies were diluted as follows and incubated at 4°*C* overnight: aggrecan (1:200, Abcam, ab36861) or collagen type II (1:100, Abcam, ab34712). Aggrecan (or collagen type II) is present when the cytoplasm was stained with a pale brown dye.

### Statistical Analysis

All the experiments were performed at least three times. The results were expressed as means ± SD. Raw statistical analysis was processed by SPSS20.0 (IBM SPSS, Chicago, IL, United States). Data were analyzed by one-way ANOVA followed by a *post hoc* test for comparison between control and treatment groups. Nonparametric data (Pfirrmann MRI grade scores) were analyzed by Kruskal–Wallis *H* test. Differences were suggested to be statistically significant when *P* < 0.05.

## Results

### Animal Surgery

There were no complications associated with the puncture operation. All experimental animals were punctured in 5 min with no prolonged bleeding. During the entire experimental period, all experimental rats were healthy like the non-puncture rats.

### Radiograph

**FIGURE 2:**
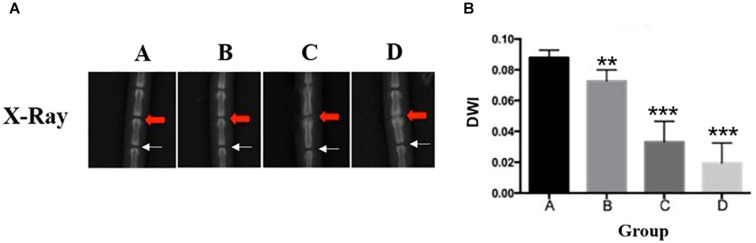
**(A)** Representative radiographs of the tail were obtained before annulus needle puncture (ANP), 4 weeks after ANP, 8 weeks after ANP, and 12 weeks after ANP (red arrow: injured Co7/8, white arrow: uninjured Co8/9). No degeneration was observed in the adjacent disks (white arrow). **(B)** The disc height index (DHI) progressively decreased over time. %DHI *post hoc* test demonstrated a statistically significant difference in DHI between pre- and post-puncture (each of the subsequent weekly measurements). **, significant difference from other group disks (*p* ≤ 0.01); ***, significant difference from other groups (*p* ≤ 0.001).

**FIGURE 3:**
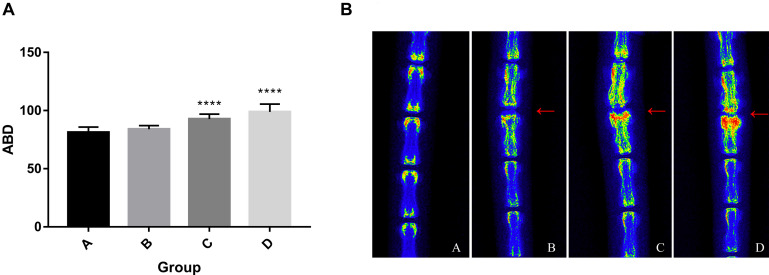
**(A)** Progressive increase of average bone density. One-way ANOVA multiple comparison showed that there was no difference between group A and group B. In both group C and group D, there were significant differences with group A (*p* < 0.001). **(B)** The heat map of representative radiographs visualized the progression of vertebral osteogenesis in punctured disc disk (red arrow) over time. ****, significant difference from other groups (*p* ≤ 0.0001)

At 4, 8, and 12 weeks post-puncture, a radiograph of the tail was, respectively, taken to assess the disk width and the ABD. The results (Figure 2A) demonstrated that the disk width progressively narrowed over time, and no disk width changes were observed in the adjacent disk of the punctured IVDs. Mean DWI was 0.088 + 0.004 at baseline, which decreased by 17.4% (*P* < 0.001) after 4 weeks of puncture, 62.3% (*P* < 0.001) after 8 weeks of puncture, and 177.9% (*P* < 0.01) after 12 weeks of puncture, respectively (Figure 2B). Notably, the punctured disk width reduced at 12 weeks. A *post hoc* analysis demonstrated a statistically significant difference in DWI value between the pre-puncture and the post-puncture weekly measurements. The ABD was, respectively, 81.20 ± 4.63 in group A, 83.93 ± 3.18 in group B, 92.65 ± 4.32 in group C, and 98.87 ± 6.69 in group D. There was no difference between group A and group B. In both group C and group D, there were significant differences with group A (*p* < 0.001 in both groups C and D; Figure 3A). In Figure 3B, a heat map of radiographs was presented to visualize the difference in bone density between groups. It is obvious that a larger red area, which meant higher bone density, was shown in groups C and D than in groups A and B.

### Magnetic Resonance Imaging

**FIGURE 4:**
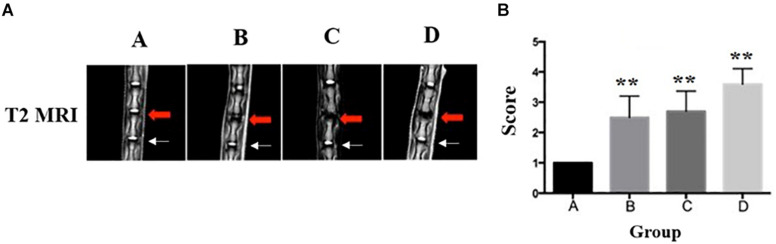
Representative T2-weighted MRI of the lumbar spine of rats in group A, group B, group C, and group D (red arrow: injured Co7/8, white arrow: uninjured Co8/9). **(A)** The disk of Co7/8 (red arrow) showed a moderate decrease of signal intensity in group B and a severe decrease of signal intensity in group C. The signal intensity in group D demonstrated whole-disk degeneration. In all groups, no degeneration was observed in the adjacent disks (white arrow). **(B)** The Pfirrmann MRI grade scores in three experimental groups (group B and group C and Group D) compared to the control group (five rats at each time point for each group). **Significant difference from other group disks (*p* ≤ 0.01).

An MRI was performed to classify the degeneration of the rat IVDs. A significantly lower T2-weighted signal intensity of MRI was found in the punctured IVDs when compared to the non-puncture IVDs (Figure 4A). Notably, no signal intensity changes were observed in the adjacent disk of punctured IVDs. In Figure 4B, the diagrams showed the Pfirrmann grades of IVDs. The RTD (Co7/8) in group A were Pfirrmann grade I, demonstrating no disk degeneration. The RTD (Co7/8) in the puncture group (group B and group C) reached Pfirrmann grade II–III and grade III–IV in group D, which indicates severe IVD degeneration.

### ANP-Induced Rat IVD Degeneration

**FIGURE 5:**
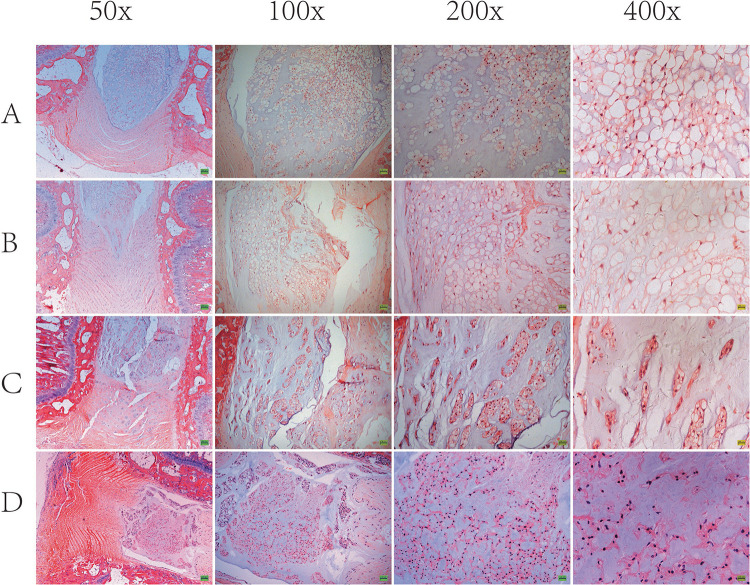
Representative H&E staining of the intervertebral disk is shown. In group A, disk (Co7/8) demonstrated well-organized, intact annulus fibrosus (AF) with concentric lamellae and notochordal cells with large vacuoles in nucleus pulposus (NP). In group B and group C and group D, disk (Co7/8) demonstrated apparent lamellar disorganization or fragmentation in AF, gradually diminished notochordal cells, and increasing chondrocyte-like cells in NP.

Based on the results of imaging experiments, we explored the effects of ANP on intervertebral disk cells using H&E staining. As shown in Figure 5, the AF in group A was arranged in an orderly fashion. However, lamellar disorganization of the annulus was observed, and few annulus fibrosus cells were detected at 4 weeks. In groups C and D, the disks distinctly revealed lamellar disorganization, with a wavy appearance and ruptured pattern fibers of the AF. Compared with the control group, the punctured disks demonstrated a loss of a clear border between the AF and NP as well as contained a low density of cells. Within the NP of punctured disks, there was a loss of vacuolated cells on H&E staining. In groups C and D, the notochord cells in NP were shown to gradually diminish, and fibrous cells increased. Thus, these results suggest that puncture-induced disk degeneration in the rat model of RTD.

### ANP-Induced Cellular Apoptosis

**FIGURE 6:**
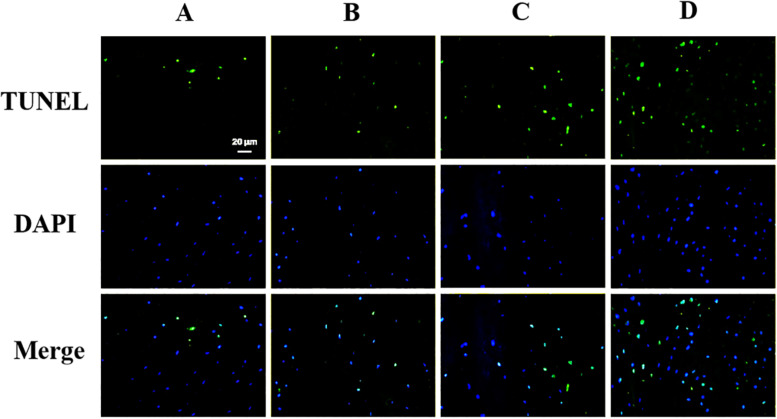
TUNEL assay was performed to evaluate the apoptosis in nucleus pulposus cells (original magnification, × 400; scale bar, 20 μm.

The apoptosis of the notochord cell in NP was evaluated by TUNEL assay staining (Figure 6). As shown in Figure 6, we observed that apoptosis of the notochord cell was significantly increased in the punctured groups compared to the control group at 8 and 12 weeks post-puncture. Compared with the ANP groups, no positive staining was observed in the non-puncture group. Thus, we conclude that ANP leads to apoptosis of the notochord cell.

### ANP-Induced Cellular ECM Degeneration

**FIGURE 7:**
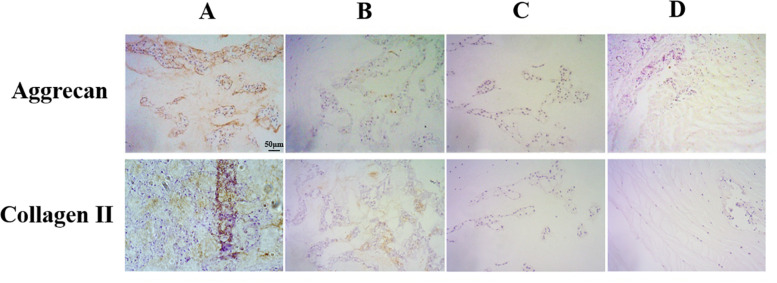
Immunohistochemical staining of aggrecan and collagen II expression in the nucleus pulposus samples (original magnification, ×200; scale bar, 50 μm). The expression of aggrecan and collagen II significantly decreased in group B and group C and group D.

Extracellular matrix (ECM) destruction, induced by inadequate anabolism, is considered as one of the characteristics representing degeneration in notochord cells. In our study, the expression levels of the ECM component collagen II proteins and aggrecan proteins were analyzed using immunohistochemical staining. As shown in Figure 7, the results showed a significant reduction of aggrecan and collagen II protein levels in the punctured groups compared to the non-puncture group. Thus, these results suggested that puncture could induce ECM degeneration in the notochord cell.

## Discussion

Intervertebral disk degeneration was often accompanied by IVD structural changes, of which the annular tear was widely accepted as a common condition to destroy the immune privilege of the disk (Ishikawa et al., 2018; Moriguchi et al., 2018). In this study, we established a novel rat ANP model in a standardized method to explore the effect of annulus fibrosus micro-injury on IDD progressive development. Unlike other models, this standard modeling was induced by a customized puncture device using 3D printing to simulate the radioactive tear injury of human AF. This customized puncture device was designed for the following advantages: (1) there were four localization holes to visualize the vessel markers, which could avoid extra bleeding from vessel injury; (2) there were four other holes between the localization holes for an accurate puncture in different orientations; (3) the puncture needle was chosen in a thinner size of 27G for micro-injury and customized to a relative depth to ensure the same puncture effect among experimental animals; and (4) the puncture needle could penetrate almost the entire layer of the AF without directly destroying the nucleus pulposus, which was ensured by a stop at the end of the needle matching the puncture holes. According to our preliminary study, the width of the NP was kept stable at less than 5 mm. We customized the length of the needle to match a customized ring; thus, the needle tip in all ANP devices was made constant at 5.5 mm. As long as the ring matched the tail and the needles subsequently matched the ring, a precise puncture without penetrating the NP could be made in all experimental segments, no matter how the tail width varied.

In normal IVDs, the extracellular matrix of the NP is mainly composed of proteoglycans to ensure NP as a water-rich tissue which can be detected by MRI T2-weighted imaging as a hyperintensity signal within the NP area. The MRI T2 imaging results showed that the lower signal intensity of the NP area was observed in the punctured disk, and Pfirrmann grading revealed that the decreasing signal intensity was time independent and slowly progressive.

Aggrecan proteoglycan and collagen II represent the ability of ECM synthesis and play an essential role in the physiological function and maintenance of disks. As shown in Figure 7, we found that ANP caused a decreased level of collagen II expression and aggrecan expression in the notochord cell, which meant a homeostasis disruption in NP tissue.

Intervertebral disk cell apoptosis has been proven as one of the most important causes of IDD (Presciutti et al., 2014). It was reported that abnormal loading or oxidative stress may promote cell apoptosis and the significant decrease of the synthesis of extracellular matrix (Ha et al., 2006; Zhao et al., 2006). Our study revealed that AF radial fissure could contribute to the IDD. We speculated that the microinjury puncture of the AF affected the uniform distribution of load stress throughout the IVD because maintaining the load stress is based on the intact AF. We assumed that the injury of the entire layer of the annulus fibrosus may cause the destruction of the oxygen partial pressure balance in the NP, which could induce apoptosis in the notochord cell. In our following study, we will use this ANP model for further research on the apoptosis mechanism to validate this speculation.

Interestingly, the radiograph results showed a time-independent increase of vertebral bone density in the punctured disk, which may imply that vertebral osteogenesis was related to the IDD. The heat map of the radiographs directly visualized the progressive osteogenesis along with the decrease of disk width. In general, the disruption of IVD homeostasis due to the destruction of the IVD structure may lead not only to progressive IDD but also to osteophyte formation of IVD. There are few previous studies in which an animal model was used for researching osteogenesis following the IDD. Thus, our study built a novel animal model to provide a potential application for the correlation research between IDD and ectopic bone formation.

## Conclusion

A standard progressive IDD rat model could be established by micro-injury IVD puncture using a novel 3D printing device. This animal model provided a potential application for research on progressive osteogenesis following IDD development.

## Data Availability Statement

The original contributions presented in the study are included in the article/supplementary material, further inquiries can be directed to the corresponding author/s.

## Ethics Statement

The animal study was reviewed and approved by the Animal Care and Use Committee of Ningbo University.

## Author Contributions

DX, LG, JL, and JT contributed to the conceptualization, project administration, funding acquisition, and reviewing and editing the manuscript. MY, XY, and WH contributed to the investigation, analysis, validation, and writing the original draft of manuscript. CZ, BH, TG, XW, and JX contributed to the investigation and analysis. All authors contributed to the article and approved the submitted version.

## Conflict of Interest

The authors declare that the research was conducted in the absence of any commercial or financial relationships that could be construed as a potential conflict of interest.
